# High prevalence and clustering of modifiable CVD risk factors among rural adolescents in southwest Nigeria: implication for grass root prevention

**DOI:** 10.1186/s12889-015-2028-3

**Published:** 2015-07-14

**Authors:** N A Odunaiya, K Grimmer, Q A Louw

**Affiliations:** Division of Physiotherapy, Faculty of Health Sciences, Stellenbosch University, Stellenbosch, South Africa; Department of Physiotherapy, University of Ibadan, Ibadan, Nigeria; International Center for Allied Health Evidence, University of South Australia, Adelaide, Australia

**Keywords:** CVD, Modifiable, Risk factors, Adolescents, Rural Nigeria

## Abstract

**Background:**

Cardiovascular disease (CVD) is an immense global problem with serious economic and social consequences. Modifiable risk factors for CVD have been identified internationally in adolescents where early intervention programs have the potential to reduce CVD risk on individual and population levels. In developing countries such as Nigeria, little is known about the prevalence of modifiable CVD risk factors among adolescents especially in the rural areas.

**Methods:**

This paper reports on a cross-sectional survey of modifiable CVD risk factors among rural adolescents in South-West Nigeria. All 15–18 years old adolescents in all the schools at Ibarapa central local government were approached and all those who assented and consented to participate in the study were involved. A total of 1500 adolescents participated in the study. Measurements of CVD risks factors taken were; smoking, physical activity, alcohol, dietary pattern using a questionnaire developed by authors. Other CVD risk factors such as waist hip ratio and BMI were taken using standardized instruments. Data were analyzed using STATA version 12.

**Results:**

Data from 1079 adolescents (56.5 % males and 53.5 % females) were analyzed. Mean age of males was 16.4 ± 1.14 years and mean age for females was 16.29 ± 1.13 years. Adolescents showed clustering of CVD risk factors with about 72 % having between two and four risk factors. A total of 102 clustering patterns were reported. The most common clustering pattern (19.6 %) included high animal lipid and salt diet.

**Conclusion:**

There is high level and clustering of CVD risk factors among rural adolescents in Southwest Nigeria. The most common clustering pattern was biased towards dietary factors. The high prevalence of CVD risk factors among rural adolescents in Southwest Nigeria suggests that urgent primary prevention programs are required to prevent the next generation of Nigerians from suffering of CVD.

**Electronic supplementary material:**

The online version of this article (doi:10.1186/s12889-015-2028-3) contains supplementary material, which is available to authorized users.

## Background

Cardiovascular disease (CVD) is on the increase in developing countries [1] causing twice as many deaths as HIV, malaria and tuberculosis combined [2] and more prevalent in the working- age population [3] resulting in large social and economic burden. The increase in CVD burden in developing countries is largely the result of an increase in the prevalence of CVD risk factors.

National and high quality studies in CVD are sparse in Africa. In many African countries CVD is the second most common cause of death after infectious diseases, accounting for about one in every ten deaths [4] and it is the leading cause of death in those 45 years and above [5]. Most of the CVD deaths in Africa occur among younger people when compared to Europe and North America, with about half of cardiovascular diseases (CVDs) due to causes other than atherosclerosis [6]. Though the burden of CVD in Sub-Saharan Africa is currently lower compared to Europe and North America, increased urbanization and lifestyle changes may result in increase in CVD burden in future. In Nigeria, CVD is fourth among the top twenty diseases responsible for mortality [7].

People dying of CVD have major modifiable risk factors which include high blood pressure, abnormal lipids, tobacco use, excessive alcohol use, physical inactivity, obesity, unhealthy diet and diabetes mellitus [8–13]. Many of these risk factors are caused by unhealthy lifestyle and habits, as such they are sometimes referred to as lifestyle risk factors. These lifestyle risk factors which include smoking, tobacco and excessive alcohol use, poor dietary patterns and physical inactivity have been observed in adolescents and adults in both developed and developing countries [14–18].

Investigating CVD risk factors among adolescents is important because adolescence is a critical temporal window for the development of obesity in adult age [19].^.^ Dietary habits, and risky behaviors, such as smoking and drinking are experimented with and established in childhood and adolescence [20]. Furthermore researchers have advocated that children and adolescent populations should be the target for cardiovascular risk factors prevention programs [21] because lifestyle risk factors are usually learnt and established during this period. CVD prevention program are thus likely to be more effective in this subpopulation. Modifiable risk factors can be prevented, treated and controlled, hence the need for early detection of risk factors and CVD prevention programs so that adolescents adopt healthy behaviors into adulthood. This is particularly important in rural Nigerian regions where there are very limited facilities and health personnel to manage CVD.

There is paucity of information on prevalence of modifiable CVD risk factors among rural adolescents in Nigeria. Few studies have identified prevalence of CVD risk factors in urban adolescents [22, 23]. This lack of information is a barrier to effective implementation of CVD prevention program in Nigeria and particularly in the rural areas. There is a need to investigate the prevalence of modifiable CVD risk factors among rural adolescents in Southwest Nigeria in order to plan CVD prevention programs for them.

This study is the first study which seeks to investigate a wide range of modifiable CVD risk factors among rural adolescents in south west Nigeria. The study aims to investigate the prevalence, clustering and pattern of clustering of modifiable CVD risk factors such as; smoking, alcohol use, physical inactivity, unhealthy dietary behaviors such as low fruit and vegetable consumption, high animal fat/cholesterol diet and high salt consumption. Other modifiable CVD risk factors investigated include high body mass index (BMI) and abdominal obesity. We hypothesized that there will be high prevalence of modifiable CVD risk factors among male and female rural adolescents and there will be no significant difference in prevalence rate of CVD risk factors between male and female rural adolescents in Southwest Nigeria.

## Methods

### Ethical consideration

Ethical approval was obtained from Stellenbosch University Health Research Ethics Committee (No 8/09/257, 2009). Approval was obtained from local educational authority in Ibarapa central local government area of Oyo State Nigeria. A letter of approval from the local inspector of education was taken to the principals of all schools in Ibarapa Central local government, Oyo state, Nigeria where the study took place. Permission to conduct the study in the schools and to involve the adolescents in the study was obtained from the principals of various schools. The principals were informed about the study; they in turn called parent-teacher association meetings where the principals explained the research to the parents present. Principals also informed adolescents about the study at general school meetings. The principals gave written proxy informed consent in addition to verbal consent from the parents. This is culturally acceptable as principals are seen as guardians of the students. The institutional ethics committee was informed of this process and it was approved.

### Study design

A cross-sectional survey was conducted.

### Participants

Participants were aged 15–18 years. Participants had to be able to read and write either English or Yoruba and had no learning difficulties.

### Sample size calculation

The sample size of 1600 was estimated, based on a cluster design effect of 5.8, an ICC of 0.5 to adjust for prevalence estimation and 90 % power for analytical analyses. This calculation was based on an initial approximate estimate of 5,400 15–18 years old adolescents in the rural community. This sample size was calculated prior to the time of data collection. Sampling was originally planned in class clusters (i.e. whole classes selected would be invited to participate). Classes in the schools in this region generally contained adolescents of mixed age (10–20 years). Of the 5400 adolescents in the region, about 2500 adolescents attend school. All 22 schools in the region were included. Based on information from school principals regarding the large numbers of students likely to be absent from class on any day (due to truancy or illness), it was decided to invite all students aged 15–18 years at all schools in the region. Since 1500 surveys had already been printed based on the estimated sample size calculation, it was administered to all eligible participants for whom we received consent.

### Study setting

The study was conducted in all secondary schools in Ibarapa central local government. The local government comprised two villages, Igboora and Idere. This region has an estimated population of 57,000 people.

### Measurement tools

#### CVD risk factor questionnaire

A specifically developed and validated questionnaire (Additional file 1) was used to measure CVD risk factors. Data for lifestyle CVD risk factors were collected using Nigeria composite lifestyle CVD risk factors questionnaire for adolescents developed by Odunaiya et al. [24] to monitor adolescent cardiovascular health in Nigeria. The questionnaire is a 33 item profile scale with six subscales which are; demographic subscale, CVD indicators subscale, smoking subscale, alcohol subscale, physical activity subscale and nutrition subscale. The scales of measurement are nominal and ordinal scales. CVD indicator subscale sought to find out adolescents who had parents and close relatives with cardiovascular disease or receiving treatment for cardiovascular disease. Other dimension of this subscale assessed breathlessness and chest pain during rest or exercise. Smoking subscale assessed likelihood of smoking in future and current smoking with number of cigarettes in the last 30 days. Alcohol subscale assessed number of standard drinks taken at any time drinking. Physical activity subscale assessed frequency (number of times in the last week) and intensity of exercise in the last week and was categorized as low moderate and high. Nutrition subscale assessed food frequency in the last week and was categorized as low, moderate and high consumption of any food type. The intra-class correlation coefficient (ICC) was used as an estimate of reliability of the questionnaire. For the English version of the questionnaire, the ICC was 0.65 in CVD indicator section, 0.70 in alcohol section, 0.50 in smoking section, 0.46 in physical activity section and 0.3 in nutrition section. According to Fleiss 1999 [25], ICC; 0–0.2 is poor, 0.2–0.4 is fair, 0.4–0.6 is moderate and 0.6–0.75 is good and >0.75 is excellent. Smoking and physical activity were moderate while CVD subscale and alcohol subscales had good reliability. Nutrition subscale had fair reliability for the English version. In the Yoruba version: CVD indicator ICC was 0.6, smoking, 0.4 alcohol 0.8, physical activity 0.4 and Nutrition 0.7. The Yoruba version showed moderate to very good reliability using Fleiss' classification. Judging from the reliability values, the Yoruba version showed a more consistent estimate. This may be because adolescents had better understanding of questions in Yoruba language than in English language. This is expected because the adolescents in the study are rural adolescents; moreover people interpret words in their mother tongue. We used the Yoruba version of the questionnaire in the study as it had better reliability than the English version of the questionnaire (except for adolescents who specifically requested for English questionnaire). For this paper we did not explore CVD indicator subscale in detail as it was not the aim of this paper.

#### Body mass index (BMI) measurement

Weight was measured with a digital scale and height was measured with a T-bar. Subjects wore light clothing without shoes for all measurement. The weight reading was recording to the nearest kilogram. Height was recorded as the nearest centimeter. BMI was calculated using CDC BMI calculator [26] and using the population BMI percentile; ≤ 5^th^ percentile is underweight, 5^th^- ≤ 85^th^ percentile is normal weight, 85^th^ ≤95^th^ percentile is overweight and ≥95^th^ percentile is obesity.

Waist Hip Ratio; Waist circumference was measured at the point of umbilicus while hip circumference was measured at the widest point of the hip [27].

### Definition of exposures (modifiable risk factors)

The modifiable risk factors included smoking, excessive alcohol use, low physical activity level and poor nutritional factors, overweight and obesity determined by BMI and abdominal obesity determined by waist hip ratio.

The specific exposures to modifiable risk factors were defined as follows:

1. Smoking: smoking of any amount of cigarettes/tobacco in the last 30 days
2. Excessive alcohol: 5 drinks or more when drinking on a typical day when drinking
3. Low physical activity: less than five days a week with at least 60 min exercise a day
4. High animal lipid diet: Eating meat five times per week or more
5. Low vegetable diet: Not eating vegetables for at least five times per week
6. Low fruit diet: Not eating fruit for at least five times per week
7. High salt diet- High salt intake was defined as adding salt to food to which salt was already added to food during the cooking process
8. High BMI: 85th- ≤ 95th percentile was considered as overweight and ≥ 95th percentile was considered obese.
9. Abdominal obesity: There are no cut-off points for Waist Hip ratio for adolescents in Nigeria. Thus, the cut off point for adult population was used (waist hip ratio in males: ≤ 0.90 cm and females: ≤ 0.85 cm were defined as the at risk exposure [27]

### Statistical analysis

The data were descriptively analyzed using means and standard deviations. Prevalence was expressed as percentages and where appropriate, the 95 % confidence intervals (CI’s) were determined. Chi squared analysis was used to determine if risk factors differed significantly between genders (*p* < 0.05 was set as the level of significance). Pearson correlation coefficients were used to assess correlation between risk factors. All analyses were done using STATA version 12 (p <0.05 was set as the level of significance).

## Results

### Response rate

All questionnaires were returned (100 % return rate) because the adolescents were assembled in classes and supervised by one of the authors and teachers in the school to complete the questionnaires. Out of 1500 adolescent who returned the questionnaires, Weight, height, waist circumference and hip circumference were measured in 1390 adolescents. Of the 1390 adolescents who completed the objectives measurements and returned the questionnaire, data from 1079 adolescents (77.6 %) were analyzed. Therefore, data of 311 adolescents were not included in the analysis because some sections of the questionnaire were not completed (possible from adolescents who could not read and write in English or Yoruba) and some of the adolescents who completed the questionnaires were older than 18 years and the data were thus also excluded.

### Sample demographics

The sample consisted of 46.5 % males and 53.5 % females. The mean weight was 47.9 kg ±8.9 for males and 48.9 kg ± 7.6 for females. Mean BMI was 18.4 kg/m^2^ ± 2.1 kg/m^2^ for males and 19.5 kg/m^2^ ± 2.5 kg/m^2^ for females. Mean waist circumference was 68.0 cm ± 5.1 cm for males and 65.0 cm ± 4.3 cm for females. Mean Waist Hip ratio was 0.8 ± 0.04 for males and 0.78 ± 0.05 for females.

### CVD indicators

Table 1 indicates the prevalence of CVD indicators. Familial factors (relatives with CVD; relatives seeing a doctor for CVD) were notably high. The prevalence was also relatively equally distributed between males and females.

**Table 1:** CVD indicators

	Prevalence% (95 % CI)	Prevalence %	Prevalence %
		Female	Male
Get tired even when not exercising (*n* = 1079)	53.5 (50.5–56.5)	51.2 (*n* = 577)	56.1 (*n* = 502)
Chest pain when exercising (*n* = 1073)	58.0 (54.6–60.6)	55.5 (*n* = 575)	60.8 (*n* = 498)
Difficult breathing after little exercise (*n* = 1074)	59.8 (56.6–62.4)	56.9 (*n* = 575)	63.1 (*n* = 499)
Relatives with CVD (*n* = 1073)	89.1 (86.6–90.4)	89.1 (*n* = 574)	88.8 (*n* = 499)
Relatives seeing doctor for CVD (*n* = 1069)	89.5 (86.8–90.6)	90.1 (*n* = 571)	88.8 (*n* = 498)

### Modifiable CVD risk factors

Table 2 illustrates the prevalence of modifiable risk factors for the sample. High salt and animal lipid diet was most prevalent in the sample. Gender differences were also noted for four factors (smoking, alcohol, obesity and physical activity).

**Table 2:** Prevalence (%) of modifiable CVD risk factors among adolescents by sex

CVD risk factors	Prevalence % (95% CI)	Prevalence % (95% CI)	Prevalence % (95% CI)
	Group	Male	Female
Smoking/tobacco^a^	7.14 (5.59–8.68)	10.2 (7.5–12.8)	4.5 (2.8–6.2)
Excessive alcohol use^a^	10.2 (8.3–12.0)	16.3 (13.1–19.6)	4.9 (3.1–6.6)
Low fruit diet	8.4 (6.7–10.1)	10.4 (7.7–13.0)	6.8 (4.7–8.8)
Low vegetable diet	6.0 (4.6–7.4)	6.8 (4.6–8.9)	5.4 (3.5–7.2)
High salt diet	65.7 (62.9–68.6)	63.0 (58.8–67.2)	68.3 (64.5–72.1)
High animal lipid diet	59.6 (56.7–62.5)	61.2 (56.9–65.4)	58.2 (54.2–62.3)
High BMI^a^	15.1 (12.9–17.2)	15.0 (11.8–18.1)	15.2 (12.3–18.2)
Abdominal obesity	3.7 (2.6–4.8)	1.8 (0.6–3.0)	5.4 (3.5–7.2)
Low physical activity^a^	27.9 (25.2–30.6)	21.9 (18.3–25.5)	33.1 (29.3–37.0)

^a^significant gender differences

### Clustering of risk factors

A total of 1029 participants had one or more risk factor. The most common single risk factor was “high salt” intake (9.3 %). The mean number of risk factors per participant was 2.1 (SD 1.1) for the group. Among females the mean number of risk factors was 2.1 (95 % CI’s 1.6–2.2) and among boys it was 2.1 (95 % CI’s 1.9–2.2). There was not a significant gender difference. Only 4.6 % of the subjects reported no risk factors and 24.1 % reported one risk factor (Table 3).

**Table 3:** Prevalence of number of risk factors per adolescent (*n* = 1079)

Number of risk factors	Prevalence (%) Group	Prevalence (%) Girls	Prevalence (%) Boys
0	4.6	4.3	5.0
One	24.1	23.1	25.3
Two	39.0	39.5	38.4
Three	23.7	24.3	23.1
Four	6.5	6.8	6.1
Five	1.9	1.6	1.8
Six	0.3	0.5	0.2

A total of 102 risk factor patterns were reported (patterns consisted of at least two risk factors), indicating wide individual variability in CVD risk profiles. The five most common clustering patterns are presented in Table 4. The five most common clustering patterns account for about 35 % of the participants.

**Table 4:** Most five common risk factor clustering patterns within the total sample (*n* = 1079)

Risk factor clustering	Prevalence (%)
High animal lipid, high salt	19.6 %
High animal lipid, high salt, high BMI	6.1 %
High animal lipid, high salt, low physical activity	4.2 %
High salt, low physical activity	3.9 %
High animal lipid diet, low physical activity	2.6 %

### Correlation between risk factors

The correlations between most risk factors were weak. Compared to any other two risk factors, there was a stronger correlation between smoking and alcohol as well as between low vegetable and low fruit intake (Table 5).

**Table 5:** Correlation between risk factors

	Smoking	Alcohol	Low fruit	Low vegetable	High salt	High animal lipid	High BMI	Abdominal obesity	Low physical activity
Smoking									
Alcohol	0.31								
Low fruit	0.01	0.01							
Low vegetable	0.01	−0.01	0.26						
High salt	0.09	−0.08	−0.01	−0.00					
High animal lipid	0.0	0.02	−0.17	−0.20	−0.06				
High BMI	−0.08	−0.04	0.03	−0.03	−0.03	−0.00			
Abdominal obesity	−0.05	−0.03	−0.04	−0.05	0.04	0.02	0.03		
Low physical activity	−0.01	−0.04	0.13	0.18	−0.10	−0.22	−0.01	0.01	

## Discussion

This is the first study of rural adolescents in Nigeria to establish the prevalence of a wide range of modifiable CVD risk factors. The findings showed adolescents have a wide range of unique clustering patterns and the most common clustering pattern was a high animal lipid and salt diet.

### Demographics

We have captured about 60 % of the school going adolescent population from this rural region on Nigeria. However, since some rural adolescents do not attend schools, our findings may not be applicable to adolescents who are not attending schools. Regarding adolescents attending schools, we think, adolescents who did not participate might not have provided very different responses because the rural adolescents in our study had similar socio-economic backgrounds and environmental exposures.

More females participated in the study than males. This is because absenteeism from school was more common among male adolescents resulting in more females being available to participate in the study. Reasons for male absenteeism at school were not explored in this study. Many questionnaires were not used for analysis because they were over 18 years. Many of the adolescents in senior classes were over 18 years because many rural adolescents start school late and some have academic challenges. Therefore they spend more time in high school than expected. Implications of this problem is not within the scope of this study.

### CVD indicators

Many adolescents in this study had CVD indicators such as chest pain during exercise and chest pain even at rest. Many of the adolescents had close relatives seeing a doctor for CVD and this could possibly indicate that familial factors may play a role. It is also important to note that chest pain could hinder the participation in physical activity. Though CVD indicators are not the focus of this paper, it will be explored in detail in a related paper.

### Clustering of CVD risk factors

The majority of adolescents had more than one CVD risk factor. This is a very high prevalence of clustering of CVD risk factors among these rural adolescents. Clustering of CVD risk factors here refers to adolescents having more than one risk factor. Clustering of CVD risk factors exposes an individual to a greater risk of CVD than having a single risk factor. The presence of clustering of risk factors for CVD indicates the need for concerted efforts for reduction and prevention of CVD risk factors among these rural adolescents. According to Commerford and Mayosi [28, 29], low prevalence of CVD in Africa in 2006 presented a unique opportunity for primordial prevention of CVD in Africa. Presently CVD is increasing in Africa and atherosclerotic risk factors are increasing both in certain rural and urban areas [29], however rural people especially adolescents could still benefit from primordial and primary prevention.

It is important to note that a wide range of clustering patterns was reported. This may imply that the clustering patterns are still developing during adolescence and that more consistent and persistent patterns will emerge at a later stage as the adolescents grow into adulthood.

### Nutritional clustering pattern

Poor dietary pattern was the most prevalent CVD risk factor observed among the adolescents in this community. A diet consisting of high animal fat diet and salt consumption was the most common clustering pattern. Adolescents in this study have established poor dietary patterns as seen in other studies [30, 31]. In the past, rural dwellers in Nigeria had good dietary patterns compared to the city dwellers. The findings from this study show that young people are no longer continuing with cultural and local dietary pattern probably because of urbanization. Adverts from media promote western diets and young people may think this pattern of feeding is associated with civilization and affluence. High salt intake is a risk factor to hypertension and food rich in animal lipid is a major risk factor for developing CVD. Many of these adolescents might migrate to urban areas either for studies or for better employment opportunities. Hence, their diets will contain even higher levels of animal lipids and salt. There is therefore the need for CVD prevention programs before urbanization.

### Current smoking

The prevalence of current smoking observed in this study is low compared to the ones observed in advanced countries [12–14]. This is encouraging though one in every ten adolescent males is a smoker (Table 2). The majority of the adolescents have tried smoking at one time with more adolescents males trying smoking than females and some may have the intention to smoke more in the future. This intention to smoke could be influenced by advertisements in the media where smoking is associated with stardom and rural adolescents feel smoking is one of the ways of showing greatness and affluence. This implies that adolescents in this study may smoke once they have the opportunity. This finding supports the findings of Muula and Mpabulungi [32] who observed that many young people are picking up the smoking habit in Africa. The fact that few of the adolescents are current smokers in this study may be due to poverty, as the majority has tried smoking and some intend to smoke in the future. There is a need for an educational program to educate rural adolescents in Nigeria about the dangers of smoking. This we believe could be built into CVD prevention programs for rural adolescents.

### Alcohol

The excessive alcohol use among these adolescents was low compared to data from advanced countries [33, 34]. This could be because hazardous drinking brings stigmatization and it is against cultural values especially in the south west. However, there is a high level of poverty in this rural community, as such this may affect the level of alcohol consumption. However, the finding implies that one in every ten adolescents consumes alcohol at a level that is detrimental to health. This calls for concerted effort to address the issue and prevent further problems.

### Physical activity

Many of the adolescents had low levels of physical activity. This is in agreement with WHO findings which show that less than one third of adolescents globally are active enough to safeguard their future health. It also supports findings among US adolescents [35, 36]. It also corroborates the study among suburban adolescents in Nigeria [14]. It is believed generally in Nigeria, without empirical data, that the rural people are adequately physically active. This finding negates this belief. Low physical activity observed in this study is quite high though not as high as observed in developed countries. Low physical activity observed in this study may be enhanced by school curriculum, evident in lack of physical education in many schools and the adolescent lifestyle of hours spent in watching TV and playing video games with no planned/voluntary participation in physical activity program. Physical education in schools needs enhancement through national school policy as done in developed countries and even some developing countries. There is a need to explore why physical education is not included in the school time table and where there is physical education on the time table, why there is no implementation. High rates of CVD are inevitable unless there is an urgent prevention program put in place.

### Obesity

Prevalence of overweight or obesity among our study participants was low compared to findings from developed countries [11–13]. While obesity is low now among these rural adolescents, with sedentary living and poor dietary pattern of high animal fat diet and fried food preference, given a short time and poverty alleviation, obesity might become a serious concern as it is in advanced countries now, therefore prevention programs are needed. More females were obese. This corroborates studies which report prevalence of overweight and obesity more in females than males [37, 38]. It is important to note that women are expected to be fat as a sign of good health and good nourishment in many rural areas in Nigeria; therefore many rural Nigerians are not likely to see overweight and moderate obesity as a problem. This calls for health education for rural adolescents.

### Abdominal obesity

Abdominal obesity was observed in this study. Abdominal obesity has been found to be strongly associated with CVD in previous studies [39, 40]. Some of the adolescents had waist hip ratio above normal. This implies prevalence of abdominal obesity among this rural adolescents. It is however, surprising to see that adolescents who have normal weight and even underweight had abdominal obesity. This could be protein malnutrition but high calorie resulting in storage of excess fat and calorie in the abdomen. Abdominal obesity predicts CVD risk.

### Prevalence rate of CVD risk factors between male and female adolescents

Excessive use of alcohol and smoking were significantly higher in male adolescents than female adolescents while obesity and physical inactivity were significantly higher in female adolescents than male adolescents. The finding from this study on smoking prevalence and sex contradicts findings from GYTS study which observed more smoking among female than male adolescents in Ibadan, a city in south west Nigeria [41]. This could be because this study was conducted among rural adolescents who may still be influenced strongly by cultural values in contrast to the adolescents in the GYTS study who live in urban areas and are becoming more westernized in their lifestyle. In Nigeria women are not expected to engage in drinking, in fact it is almost a forbidden thing for a woman to drink alcohol in public places like restaurants especially in rural areas. Smoking is also associated with sex because women are virtually forbidden to smoke; smoking in women is linked with social vices such as prostitution. This may be the reason for alcohol and smoking being significantly higher among male gender in this study. However, it is important to note that smoking and alcohol did not have 0 % prevalence among female adolescents. This implies that young girls even in rural Nigeria are dropping some traditional beliefs and picking up some westernized lifestyle. Low physical activity and obesity were significantly higher in females. This may be because women are expected to be fat and girl child in rural Nigeria grows up believing that she needs to be fat. Also low physical activity was significantly associated with female adolescents. The need for holistic and comprehensive and gender related CVD prevention program in rural Nigeria is indicated.

### Limitations

Future research should also involve rural adolescents who are not attending schools and those who attend school but are not proficient in English or a local language to complete the questionnaires. This will improve the generalizability of the findings. All responses were self-reported in our study. Risk factors such as salt intake may need a different strategy. In Nigeria, it is routine to add salt while preparing food. However, future studies may need to engage parents or caregivers who prepare food to obtain more insight regarding the amount of salt added. In addition, we also did not consider salt included in processed food. Another limitation was that we did not interpret any “animal lipid” as lean. Although consumption of lean meat in Nigeria is uncommon, it should be considered in future research. In our study, we did not measure level of physical activity (or level of fitness) objectively and this is recommended in future studies. Although we have tested the psychometric properties of the questionnaire, we did not test reliability of the objective measures and this should be done in future research. In addition, for our study we included the use of adult cut off for abdominal obesity.

## Conclusion

CVD risk factor clustering is common among rural adolescents. A high salt and animal fat diet is the most prevalent CVD clustering pattern among rural adolescents who attend school. Although smoking and alcohol was prevalent among this sample of rural Nigerian adolescents, it is less prevalent than in developed countries. There is a need to develop, implement and trial primordial and primary prevention programs in rural areas. While all CVD factors should be addressed, such programs should consider dietary aspects as a priority focus for these rural, Nigerian adolescents. We recommend that similar studies be conducted in other geopolitical zones of Nigeria considering that Nigeria is multiethnic and diets differ between zones.

## Additional file

Additional file 1:
**Nigeria composite lifestyle cvd risk factors questionnaire for adolescents.**
