# Supplementary material for: High prevalence and clustering of modifiable CVD risk factors among rural adolescents in southwest Nigeria: implication for grass root prevention
Source: BMC Public Health. 2015 Jul 14;15:661. doi: 10.1186/s12889-015-2028-3 (PMC4501296; doi:10.1186/s12889-015-2028-3)
Supplement: Additional file 1: — Nigeria composite lifestyle cvd risk factors questionnaire for adolescents. [file 12889_2015_2028_MOESM1_ESM.pdf]

NIGERIA COMPOSITE LIFESTYLE CVD RISK FACTORS QUESTIONNAIRE FOR ADOLESCENTS

Thank you for agreeing to complete this questionnaire which is about health behaviours that can cause heart disease. We have developed it to get information from you about things you do that may affect your heart health.

The information we are getting from you will be used to develop programs that will help young people like you. There are no wrong and right answers, so answer the questions based on what you know.

The answers you give will be kept secret and will only be used for research purpose.

Kindly read every question thoroughly and tick the right box. Use a pencil so you can erase any answer you tick by mistake.

INSTRUCTIONS FOR COMPLETING QUESTIONNAIRE

- **Use HB pencil only**
- **Tick your answer in the appropriate box.**
- **Where required, complete on dotted line provided.**
- **If you change your answer, erase your old answer completely.**
- **Each section deals with a different aspects such as personal details, etc.,**

SECTION 1
Personal information

1. Gender: ☐ **Male** ☐ **Female**

2. Date of Birth: **…………(yyyy)/……(mm)/……(dd)**

3. How old are you ?

- ☐ 12 years old or younger
- ☐ 13 years old
- ☐ 14 years old
- ☐ 15 years old
- ☐ 16 years old
- ☐ 17 years old
- ☐ 18 years old or older

4. In what class are you?

- ☐ JSS3 grade
- ☐ SSS1 grade
- ☐ SSS2 grade
- ☐ SSS3 grade
- ☐ Other grades

SECTION 2
CVD indicators and family history of CVD

5. Do you get tired easily even when you don't do exercise? Yes ☐ No ☐

6. Do you have chest pain when you do exercises such as running, playing football, pounding? Yes ☐ No ☐

7. Do you find it difficult to breathe after a little exercise such as climbing stairs, walking about for 10 minutes? Yes ☐ No ☐

8. Do either of your parents or close relation have heart disease? Yes ☐ No ☐

9. Do any of your parents or close relation receive treatment from a doctor for heart disease, hypertension or diabetes? Yes ☐ No ☐

SECTION 3
Tobacco usage

10. Answer NO if you have never smoked and do not intent to smoke in future (else answer YES) Yes ☐ No ☐

11. You have tried cigarette smoking, even one or two puffs Yes ☐ No ☐

12. How old were you when you smoked a whole cigarette for the first time?

- ☐ I have never smoked a whole cigarette
- ☐ 8 years old or younger
- ☐ 9 or 10 years old
- ☐ 11 or 12 years old
- ☐ 13 or 14 years old
- ☐ 15 or 16 years old
- ☐ 17 years old
- ☐ 18 years old or older

13. You have tried smoking, but not smoked in the past 30 days Yes ☐ No ☐

14. Do you intend smoking in the future? Yes ☐ No ☐

15. During the past 30 days, on how many days did you smoke cigarettes?

- ☐ 0 days
- ☐ 1 or 2 days
- ☐ 3 to 5 days
- ☐ 6 to 9 days
- ☐ 10 to 19 days
- ☐ 20 to 29 days
- ☐ All 30 days

16. During the past 30 days, on the days you smoked, how many cigarettes did you smoke per day?

- ☐ I did not smoke cigarettes during the past 30 days
- ☐ Less than 1 cigarette per day
- ☐ 1 cigarette per day
- ☐ 2 to 5 cigarettes per day
- ☐ 6 to 10 cigarettes per day
- ☐ 11 to 20 cigarettes per day
- ☐ More than 20 cigarettes per day

17. You have smoked cigarettes daily, that is, at least one cigarette every day for 30 days? Yes ☐ No ☐

18. During the past 30 days, on how many days did you use **chewing tobacco, snuff or smoke raw tobacco?**

- ☐ 0 days
- ☐ 1 or 2 days
- ☐ 3 to 5 days
- ☐ 6 to 9 days
- ☐ 10 to 19 days
- ☐ 20 to 29 days
- ☐ All 30 days

SECTION 4
Alcohol usage

The next 4 questions ask about drinking alcohol. This includes drinking beer, wine, and hot drink such as ogogoro. For these questions, drinking alcohol does NOT include drinking a few sips of wine for religious purposes.

19. How often do you have a drink containing alcohol?

- ☐ Never
- ☐ Monthly
- ☐ 2 - 4 times a month
- ☐ 2 - 3 times a week
- ☐ 4 or more times a week

20. How old were you when you had your first drink of alcohol (more than a few sips)?

- ☐ I have never had a drink of alcohol other than a few sips
- ☐ 8 years old or younger
- ☐ 9 or 10 years old
- ☐ 11 or 12 years old
- ☐ 13 or 14 years old
- ☐ 15 or 16 years old
- ☐ 17 years old or older

21. How many standard drinks of alcohol (1 standard drink is about 350 ml or equivalent of one bottle of small coke) do you have on a typical day when drinking?

- ☐ 1 or 2
- ☐ 3 or 4
- ☐ 5 or 6
- ☐ 7 to 9
- ☐ 10 or more

22. How often do you have six or more drinks on one occasion?

- ☐ Never
- ☐ Less than monthly
- ☐ Monthly
- ☐ Weekly
- ☐ Daily or almost daily

SECTION 5
Physical activity

23. During the past 7 days, on how many days were you physically active for a total of **at least 60 minutes per day**? (Add up all the time you spend in any kind of physical activity that increases your heart rate and makes you breathe hard some of the time.)

- ☐ 0 days
- ☐ 1 day
- ☐ 2 days
- ☐ 3 days
- ☐ 4 days
- ☐ 5 days
- ☐ 6 days
- ☐ 7 days

24. Which of the following activities have you done during the past seven days?

	0	1-2	3-4	5-6	7 or more
Skipping	☐	☐	☐	☐	☐
Rowing, canoeing	☐	☐	☐	☐	☐
Brisk walk	☐	☐	☐	☐	☐
Bicycling	☐	☐	☐	☐	☐
Jogging or running	☐	☐	☐	☐	☐
Aerobics	☐	☐	☐	☐	☐
Swimming	☐	☐	☐	☐	☐
Baseball or soft ball	☐	☐	☐	☐	☐
Football	☐	☐	☐	☐	☐
Dance	☐	☐	☐	☐	☐
Badminton	☐	☐	☐	☐	☐
Volley ball	☐	☐	☐	☐	☐
Table tennis	☐	☐	☐	☐	☐
Lawn tennis	☐	☐	☐	☐	☐
Basket ball	☐	☐	☐	☐	☐
Soccer	☐	☐	☐	☐	☐
Cricket	☐	☐	☐	☐	☐
Gardening /farming	☐	☐	☐	☐	☐
Others	☐	☐	☐	☐	☐

25. On an average school day, how many hours do you watch TV?

- ☐ I do not watch TV on an average school day
- ☐ Less than 1 hour per day
- ☐ 1 hour per day
- ☐ 2 hours per day
- ☐ 3 hours per day
- ☐ 4 hours per day
- ☐ 5 or more hours per day

26. On an average school day, how many hours do you play video or computer games or use a computer or handset for something that is not school work? (including activities such as computer games, and the Internet.)

- ☐ I do not play video or computer games or use a computer for something that is not school work
- ☐ Less than 1 hour per day
- ☐ 1 hour per day
- ☐ 2 hours per day
- ☐ 3 hours per day
- ☐ 4 hours per day
- ☐ 5 or more hours per day

27. Do you have Physical education (PE) practical in your school time table? Yes ☐ No ☐

28. In an average week when you are in school, on how many days do you go to Physical Education (PE) practical classes?

- ☐ 0 days
- ☐ 1 day
- ☐ 2 days
- ☐ 3 days
- ☐ 4 days
- ☐ 5 days

29. During the past 12 months, on how many sporting competition/inter house sport did you take part? (Example; football, sprint, relay)

- ☐ 0 teams
- ☐ 1 team
- ☐ 2 teams
- ☐ 3 or more teams

SECTION 6
Nutritional information

30. How many times a week do you eat each food on this list below?

	Days per Week						
	1	2	3	4	5	6	7
**MEAT**							
Pork							
Turkey							
Liver							
Kidney							
Other organ meat (intestine, etc)							
Meat pies/pastries							
Ready meals							
Beef (cow meat)							
Goat							
Chicken							
**BREAKFAST CEREALS**							
Pap (from millet, guinea corn etc							
Golden morn							
Corn flakes							
Any other brands							
**VEGETABLES**							
Salad vegetables							
Leafy green vegetables (E.g. green, ugwu, waterleaf, ewedu)							
Green peas							
Carrot							
Pepper							
Baked beans							
Beans							
Soya beans							
Other							
**FRUITS**							
Garden eggs							
Oranges							
Bananas							
Grape fruits							
Pawpaw							
Mangoes							
Cashew							
Apples							
Other							
**Other FOOD**							
Cassava and cassava products							
Yam and yam product							
Plantain							
Bread							
Eggs							
Fish							
Ice cream							
Rice							
Pasta/Noodles/Spaghetti							

31. Do you add salt to food at table if salt was already added to food during the cooking process? Yes ☐ No ☐

32. How much salt do you eat compared to other young people like you?

- ☐ More salt
- ☐ Less salt
- ☐ Same amount of salt

33. How do you prefer your food cooked? (E.g meat, fish, egg, plantain)

- ☐ Boiled
- ☐ Fried
- ☐ Roasted

This is the end of the survey.

Thank you !!
